# Oxidative Stress and Proinflammatory Cytokines Contribute to Demyelination and Axonal Damage in a Cerebellar Culture Model of Neuroinflammation

**DOI:** 10.1371/journal.pone.0054722

**Published:** 2013-02-19

**Authors:** Alessandra di Penta, Beatriz Moreno, Stephanie Reix, Begoña Fernandez-Diez, Maite Villanueva, Oihana Errea, Nagore Escala, Koen Vandenbroeck, Joan X. Comella, Pablo Villoslada

**Affiliations:** 1 Center of Neuroimmunology, Institute of Biomedical Research August Pi Sunyer (IDIBAPS) – Hospital Clinic of Barcelona, Barcelona Spain; 2 Neurogenomiks, University of the Basque Country (UPV/EHU), Leioa, Spain; 3 Vall d'Hebron Institut de Recerca (VHIR), Universitat Autonoma de Barcelona and CIBERNED, Barcelona, Spain; 4 IKERBASQUE, Basque Foundation for Science, Bilbao, Spain; Hannover Medical School, Germany

## Abstract

**Background:**

Demyelination and axonal damage are critical processes in the pathogenesis of multiple sclerosis (MS). Oxidative stress and pro-inflammatory cytokines elicited by inflammation mediates tissue damage.

**Methods/Principal Findings:**

To monitor the demyelination and axonal injury associated with microglia activation we employed a model using cerebellar organotypic cultures stimulated with lipopolysaccharide (LPS). Microglia activated by LPS released pro-inflammatory cytokines (IL-1β, IL-6 and TNFα), and increased the expression of inducible nitric oxide synthase (iNOS) and production of reactive oxygen species (ROS). This activation was associated with demyelination and axonal damage in cerebellar cultures. Axonal damage, as revealed by the presence of non-phosphorylated neurofilaments, mitochondrial accumulation in axonal spheroids, and axonal transection, was associated with stronger iNOS expression and concomitant increases in ROS. Moreover, we analyzed the contribution of pro-inflammatory cytokines and oxidative stress in demyelination and axonal degeneration using the iNOS inhibitor ethyl pyruvate, a free-scavenger and xanthine oxidase inhibitor allopurinol, as well as via blockage of pro-inflammatory cytokines using a Fc-TNFR1 construct. We found that blocking microglia activation with ethyl pyruvate or allopurinol significantly decreased axonal damage, and to a lesser extent, demyelination. Blocking TNFα significantly decreased demyelination but did not prevented axonal damage. Moreover, the most common therapy for MS, interferon-beta, was used as an example of an immunomodulator compound that can be tested in this model. *In vitro,* interferon-beta treatment decreased oxidative stress (iNOS and ROS levels) and the release of pro-inflammatory cytokines after LPS stimulation, reducing axonal damage.

**Conclusion:**

The model of neuroinflammation using cerebellar culture stimulated with endotoxin mimicked myelin and axonal damage mediated by the combination of oxidative stress and pro-inflammatory cytokines. This model may both facilitate understanding of the events involved in neuroinflammation and aid in the development of neuroprotective therapies for the treatment of MS and other neurodegenerative diseases.

## Introduction

Axonal damage is a critical process in the pathogenesis of several chronic brain diseases, including neurodegenerative diseases, (Alzheimeŕs disease, Parkinsońs diseases, and amyotrophic lateral sclerosis) inflammatory diseases (multiple sclerosis), or acute diseases such as stroke and brain trauma [1], [2], [3]. Axonal damage is a complex process that involves alterations in multiple pathways, mitochondrial dysfunction, oxidative stress, ischemia, ATP depletion, ion channel redistribution, axonal transport impairment and reduction in trophic support [4]. These processes converge to induce Wallerian degeneration and dying-back, or axonal degeneration. By identifying the pathways that contribute to axonal damage, new avenues toward the development of neuroprotective therapies to treat brain diseases could be opened [2].

Oxidative stress is a deleterious condition that can cause cell damage, and subsequent cell death, due to oxidation of cardinal cellular components, such as lipids, proteins, and DNA [5]. Reactive oxygen species (ROS) are generated via several reactions, including the incomplete reduction of bimolecular oxygen (O_2_), resulting in the generation of superoxide (O_2_•), hydroxyl radicals (•OH) and hydroxyperoxide (H_2_O_2_). The most common cellular free radicals are the hydroxyl radical (OH·), the superoxide radical (O_2_–·), and nitric monoxide (NO·). Other molecules that are not free radicals but that can lead to the generation of free radicals through various chemical reactions include hydrogen peroxide (H_2_O_2_) and peroxynitrite (ONOO–). These ROS can be counterbalanced by natural enzymatic antioxidants (*e.g.,* superoxide dismutase, catalase) and non-enzymatic antioxidants (*e.g.,* uric acid, ascorbic acid, glutathione), which are expressed under the control of transcription factors, such as nuclear factor E2-related factor 2 (Nrf2). The central nervous system (CNS) appears to be especially vulnerable to oxidative stress due to its high rate of oxygen consumption, the abundance of iron, the low levels of molecular antioxidants, and the susceptibility of neurons or oligodendrocytes due to their particular metabolic properties [6].

The study of the contribution of different pathways to CNS damage and the effects of therapies in preventing such damage requires the development of suitable models. Here we characterize at the morphological level an in vitro model of brain inflammation by challenging cerebellar cultures with endotoxin in order to promote microglia activation. In this model, we assessed the role of oxidative stress and pro-inflammatory cytokines in producing axonal damage and demyelination as a response to microglial activation. Studies were performed in mouse cerebellar organotypic cell cultures, which provide a well-preserved structure of brain tissue including all implicated cell populations (microglia, astrocytes, neurons, axons, myelin and oligodendrocytes). We found that in response to inflammation due to microglia activation in cerebellar organotypic cultures the axons and myelin were damaged by the induction of oxidative stress and pro-inflammatory cytokines.

## Materials and Methods

### Animals and cerebellar organotypic cultures

All animal experiments were performed using C57BL/6J mice (Harlan Laboratories). Animals were handled in accordance with the European Communities Council Directive (86/609/EEC amended by Directive 2005/65/EC) and the Spanish regulations for the procurement and care of experimental animals (1201 RD/2005, October 10), and the study was approved by the Ethical Committee on Animal Research of the University of Barcelona. All possible efforts were made to minimize animal suffering and limit the number of animals used. Cerebellar slice cultures were based on previously published protocols [7], [8] and were prepared from 8-day-old C57BL/l6 mice. Cerebellum was cut using a vibratome obtaining tissue slices (350 μm thick). Three slices were plated on Millicell-CM culture inserts. Cultures were incubated at 37°C, 5% CO_2_ in 50% basal medium containing Earle's salt, 25% Hank's buffered salt solution, 25% inactivated horse serum, 5 mg/ml glucose, 0.25 mM L-glutamine and 25 μg/ml Penicillin/Streptomycin. In all experiments, cerebellar slices were maintained in culture for 7 days for reducing microglia activation and allowing cultures to myelinate before commencing the studies. After 7 days *in vitro* (DIV), cultures were treated with different concentrations of LPS (5, 10, 15 and 20 µg/ml) for 1, 3, 6, 12, 24, 48, 72 and 96 h, and then fixed in 4% paraformaldehyde (PFA) for immunofluorescence analysis, or homogenized to obtain protein extracts. Untreated control tissue (both for microscopy imaging and for molecular analysis) was incubated for identical periods of time as treated cultures.

### BV-2 culture

BV-2 cells were generously provided by Prof Antonio Celada (IRB, Barcelona, Spain) [9] and were maintained in DMEM containing 5% heat inactivated FBS, 4mM L-Glutamine (SAFC biosciences), 20 mM Hepes (Sigma) and appropriate antibiotics at 37°C in a humidified chamber with 5% C0_2_. Before treatment cells were washed twice with DMEM, then incubated 6, 12 or 24 h in 10 ml of serum-free medium containing 100 ng/ml LPS (Sigma L4391) and different concentrations of Allopurinol (100 µM or 1 mM).

### Immunofluorescence microscopy

Cerebellar slices were fixed with 4% paraformaldehyde (PFA) for 40 min, washed with PBS for 10 min, and blocked at RT for 2 h in 10% normal goat serum (NGS: Vector Laboratories, Burlingame, USA) and 0.5% Triton X-100 in PBS. The slices were incubated overnight at 4°C with the distinct primary antibodies (Table 1) in blocking solution (10% NGS and 0.3% Triton X-100 in PBS). After further washing, the slices were incubated in blocking solution containing the secondary antibody mixture prior to three washes with PBS. The secondary antibodies used were mouse IgG Cy2-linked, rabbit IgG Cy3-linked (from goat, 1∶200, GE Healthcare, Freiburg, Germany) and goat anti-rat IgG Alexa Fluor 488 (1∶200, Molecular Probes, Eugene, OR). Propidium iodide (Fluka) was used at 5 µg/ml for 2 h at 37°C and 5% CO_2_. The slices were mounted in Gel/Mount anti-fading mounting medium (Biomeda, Foster City, CA) and pictures were made by confocal scanning microscopy from single images all through the whole tissue (but avoiding the surface of the culture in contact with air) (Zeiss LSM 510). Demyelination and axonal loss were quantified as described elsewhere [10], [11]. Briefly, demyelination was quantified as the percentage of axons stained with NfL with MBP surrounding sheaths respect to the total number of axons (without MBP sheaths). Axonal loss was quantified as the percentage of axons stained with non-phosphorylated neurofilaments (SMI32) respect to the total number of axons (NfH: phosphorylated and non-phosporylated neurofilaments).

**Table 1:** List of primary antibodies used for immunofluorescence studies.

Antigen	Description	Dilution	Company
**MHCII**	Major Histocompatibility Complex class II: rat anti-mouse MHC class II (I-A) monoclonal antibody	1∶300	Chemicon
**CD11b/OX42**	mouse anti-rat CD11b	1∶150	Serotec
**NfL**	Neurofilament light C28E10, rabbit mAb	1∶500	Cell Signaling
**NfH**	Neurofilament heavy (phosphorylated and non-phosphorylated NfH): rabbit polyclonal antiserum against the 200 kD Neurofilament Heavy. Ref. Ab81351	1∶200	AbCam
**SMI32**	non-phosphorylated neurofilament heavy SMI32	1∶200	Stenberg
**MBP**	Myelin Basic Protein: rat anti-MBP (82-87) antibody	1∶200	Serotec
**NeuN**	Neuronal Nuclei: anti-NeuN mouse mAb	1∶500	Chemicon
**iNOS**	Inducible nitric oxide synthase: purified rabbit anti-iNOS/NOS type II	1∶200	BD Bioscience
**COXI**	Mitochondrial Complex IV subunit I monoclonal antibody COXI	1∶200	MitoScience
**Casp3**	Caspase 3: anti-Casp3 rabbit	1∶300	Cell Signaling
**Iba1**	Ionized calcium binding adaptor molecule 1: anti-Iba 1, rabbit	1∶400	Wako
**Nrf2**	Nrf2 (C-20)	1∶75	Santa Cruz

### Electron microscopy

The cerebellar slices were fixed for 24 h in 2% PFA and 2.5% glutaraldehyde in PBS 0.1 M at 4°C. Then, they were washed in PBS 0.1 M for 12 h before post-fixation treatment with 2% osmium tetroxide in PBS 0.1 M for 1 h at 4°C, followed by posterior dehydration and inclusion in Epoxy embedding medium (EPON). Ultra-thin sections were stained with 1% uranylacetate and lead citrate solution. Samples were observed with a Tecnai SPIRIT Transmision Electron Microscope (FEI Company, Eindhoven, The Netherlands) working at an acceleration voltage of 120 KV. Images were acquired with a Megaview III camera and digitized with the program iTEM (Soft Imaging System).

### TUNEL assay

Tissues sections, fixed with 4% PFA, were permeabilized with 1.5% Triton X-100 (in PBS) overnight at 4°C. Then the solution was removed to add sodium citrate solution (0.1% in H_2_O) 1 h at RT. The detection of cells with DNA-strand breaks was performed by the TUNEL labelling method using terminal transferase recombinant and the Fluorescein −12-dUTP (ROCHE) for 2h at 37°C. The reaction was stopped with 20 mM EGTA and the sections were washed 2 times with PBS. The labeled slides were analyzed by confocal microscopy (Olympus FV1000). In addition, for cell death quantification, nuclei were stained with 0.5 µg/mL of Hoechst 33342. Hoechst stained nuclei were scored as number of total cells and TUNEL positives cells were scored as dead.

### Active caspase-3 immunofluorescence

Cultures were rinsed with PBS at RT and fixed in 4% PFA for 30 min. They were then washed twice with PBS and permeabilized and blocked with 3% FBS and 0.1% Triton X-100 in PBS for 60 min. Cultures were incubated overnight at 4°C with rabbit polyclonal anti-cleaved caspase-3 (Cell Signaling) diluted 1∶150, rinsed three times with PBS, and incubated with Alexa Fluor 594-conjugated anti-rabbit secondary antibodies (Molecular Probes, Eugene, OR) diluted 1∶250 for 1 h at RT and protected from light. Finally, cells were stained with 0.05 μg/ml Hoechst 33258 for 30 min. For double staining Casp3/MBP the cultures were permeabilized as describe above and incubated overnight at 4°C with polyclonal anti-cleaved caspase-3 and rat anti-MBP (1∶200). Alexa Fluor 546-conjugated anti-rabbit and 488-conjugated anti-rat secondary antibodies were used.

### ELISA

Mouse cerebellar organotypic cultures were stimulated with LPS for different periods of time (0, 3, 6, 12, 24, 48, 72 and 96 hrs), or with LPS plus IFN-β (1000U/µl: Calbiochem) or Allopurinol (100 µM or 1 mM) and the culture supernatants were collected to quantify the secreted IL-1β, TNF-α and IL-6. Mouse ELISA Kits were used according to the manufacturer's instructions (eBioscience, San Diego, CA, USA).

### Western Blots

Three cerebellar slices per group were used for analysis. Western-blots were performed as previously described [12]. Briefly, total protein (10 μg) from cerebellar slices was separated by SDS-polyacrylamide gel electrophoresis, transferred onto a nitrocellulose membrane and hybridized for 2 h or overnight with primary antibodies diluted in the same blocking buffer: mouse anti-CNPase (2′,3′-cyclic-nucleotide 3′-phosphodiesterase) 1∶500 (Abcam), rabbit anti-iNOS (inducible Nitric Oxide Synthase) 1∶200 (BD Bioscience). Protein load was assessed and normalized using Ponceau S staining. Antibody binding was detected with HRP-conjugated anti-mouse or anti-rabbit secondary antibodies (Cell Signaling) used at a concentration of 1∶2,000.

### ROS Assays

ROS were assayed with H_2_DCFDA (50 µM, Invitrogen) added to the cerebellar organotypic cultures for 1 hour at 37°C. Unincorporated H_2_DCFDA was removed by washing the slices twice with PBS and the fluorescence was measured on a spectrofluorometer after excitation at 485 nm and emission at 535 nm.

### Blocking TNF-α using the recombinant Fc-TNFR1 construct

Fc-TNFR1 was produced as described previously [13]. After 7 days in culture the Fc-TNFR1 construct (Fc-TNFR1: 1/50 dilution) was added 2 h before challenging the cultures with human TNF-α at 20 and 40 ng/ml. Samples were collected 24 and 48 h after LPS stimulation, fixed in 4% PFA for 45 minutes and stored at 4°C in PBS-azide until immunostaining was performed.

### MTT assay

The MTT assay was performed 24 and 48 h after the TNFα challenge to assess cell survival in the organotypic tissue after exposure to Fc-TNFR1 and prior to the TNF-α challenge. The results provided us with the optimal concentration of Fc-TNFR1 required to block endogenous TNF-α. MTT (50 μl, final concentration 0.5 mg/ml) was added to each well and the tissue was incubated for 3 h at 37°C. The tissue was recovered in 0.1 M HCl 2-propanol, incubated again for 25 minutes at room temperature and centrifuged at maximum speed for 3 min. The supernantants were analyzed by spectrophotometer at 570 nm.

### Quantitative PCR (qPCR)

Three cerebellar slices per group were used for analysis. RNA was extracted following the manufacturer's instructions (Macherey-Nagel) and quantified with Nanodrop. RNA (100 ng) was reverse-transcribed to cDNA using random primers according to the manufacturer's instructions (Applied Biosystems). Subsequently, qPCR was performed with the Supermix for SsoFast EvaGreen (Biorad) on a 7500 Fast Real-Time PCR System (Applied Biosystems) and the qPCR QuantiTect Primer Assay were performed for each of the target genes (Qiagen). The expression of the transcripts of interest was normalized to that of endogenous HPRT1, and the data expressed relative to the mean expression in the untreated control group.

### Statistical analysis

**Figure 1:**
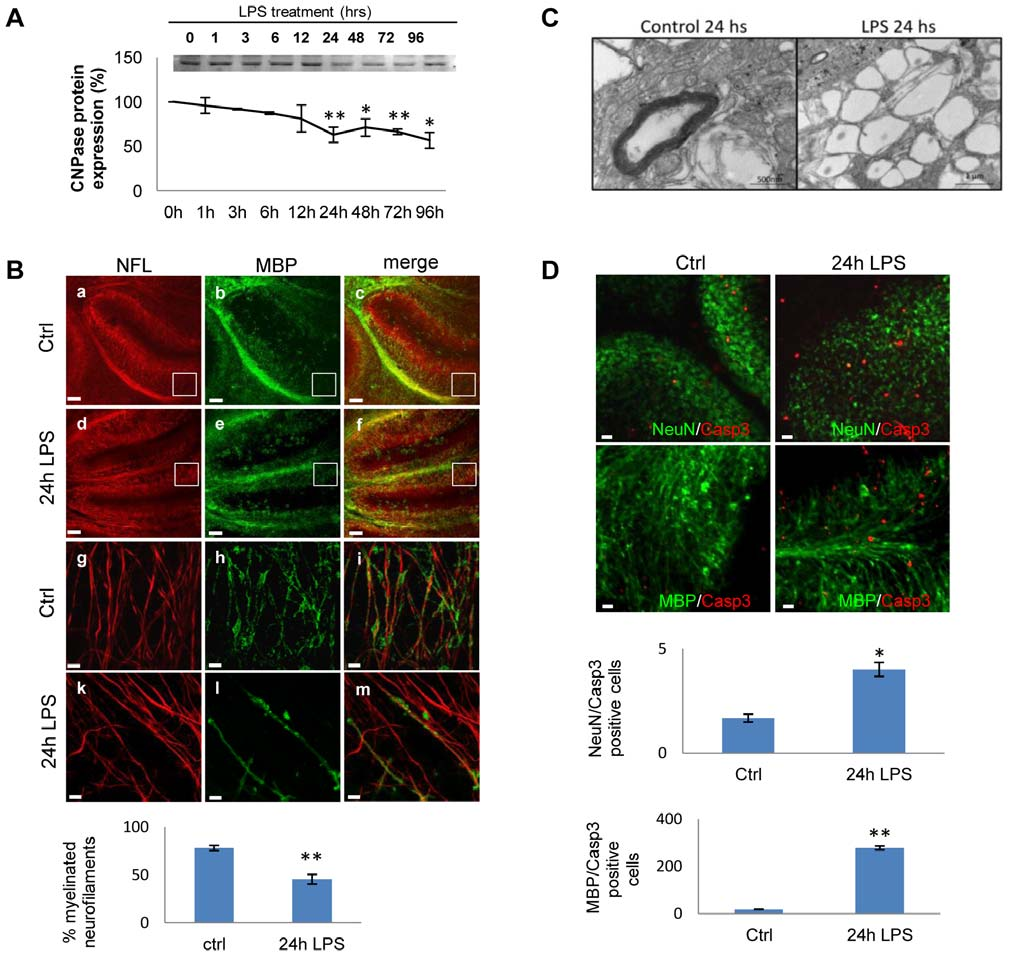
Microglial activation induces demyelination in mouse cerebellar cultures. A) Cerebellar cultures were stimulated with LPS (15 µg/ml) for different periods of time (0 to 96 h) and CNPase expression was assessed by Western-blot. Protein expression was quantified and normalized to the total protein loaded, and the results are expressed as a percentage with respect to the controls (100%). Error bars indicate the standard error. **P<0.01. B) Immunofluorescence for NfH (red) and MBP (green) in cerebellar cultures treated with LPS (15 µg/ml: panels d-f and k-m) or control slices (Ctrl, panels a-c and g-i). Panels g-m show a higher magnification (×60) of images in a-f (white boxes in panels a-f). Scale bars  = 100 µm (panels a-f) and 5 µm (panels g-m). The graph represent the percentage of myelinated axons (double staining for MBP and NfH) compared to unmyelinated axons (NfH). C) Cultures were treated with LPS for 24h and then demyelination was analyzed by electron microscopy. D) Cerebellar cultures were treated with LPS (15 µg/ml) for 24 h and then immunostained for MBP/Casp3 or NeuN/Casp3 colabeling. Scale bar  = 10 µm. The graphs represent the percentage of cell death by quantifying the co-localization of active Casp3 immunofluorescence in conjunction with MBP or NeuN staining. Student's *t-*test was used to determine statistical significance.

**Figure 2:**
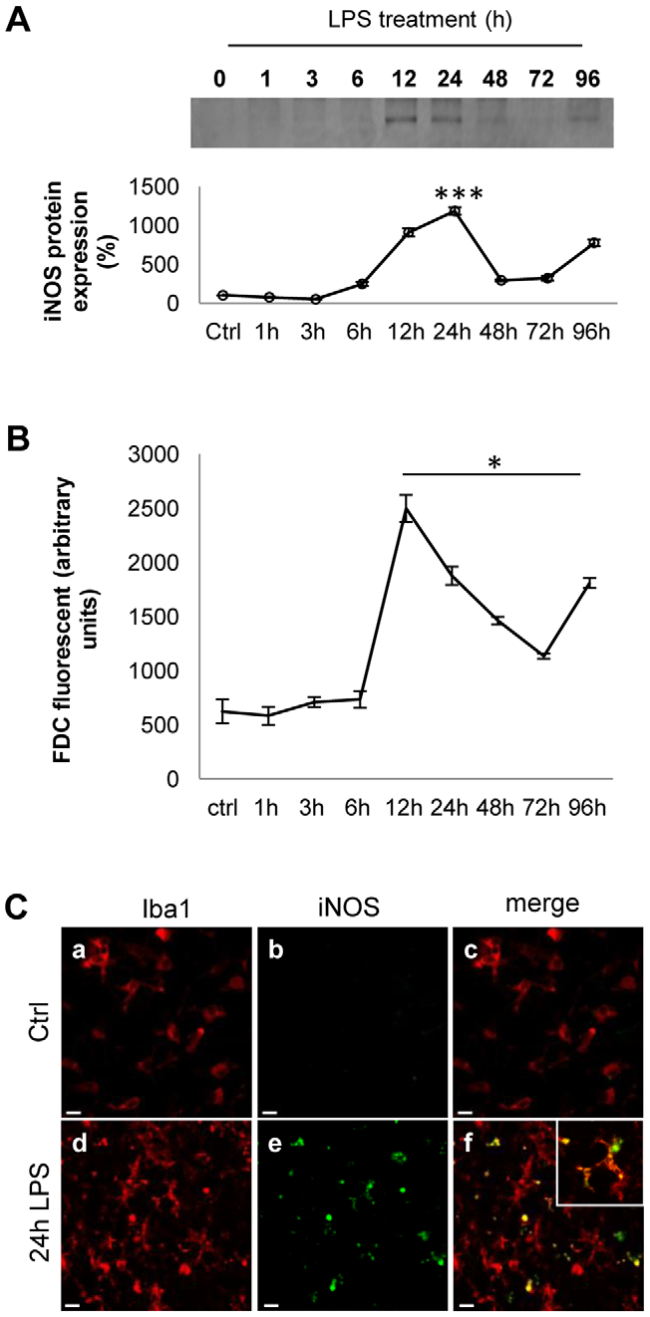
Microglial activation by LPS induces oxidative stress in cerebellar cultures. A) iNOS expression after LPS challenge: Western-blot analysis of iNOS expression in cerebellar cultures after LPS stimulation (15 µg/ml). Band intensity was calculated by densitometry and expressed as a percentage in the graph. The change in iNOS expression was calculated with respect to control (untreated cultures) and normalized with respect to total protein. Error bars indicate the standard error. ***P<0.001. B) ROS production after LPS challenge: cerebellar cultures were treated with LPS for different periods of time and ROS generation was measured by spectrofluorometry. Values in the bar graph represent arbitrary units and the error bars indicate the standard error. *P<0.05. Statistical analysis was performed using Student's *t-*test.C) Expression of iNOS by activated microglia: Cerebellar cultures were treated with LPS for 24 h and immunostainined for Iba1 (red, panels a and d) and iNOS (green, panels b and e) in organotypic cultures treated with LPS (15 µg/ml) for 24 h. Panels c and f shows the merged signals. Inset shows an enlarged image from panel f. Scale bar  = 10 µm.

**Figure 3:**
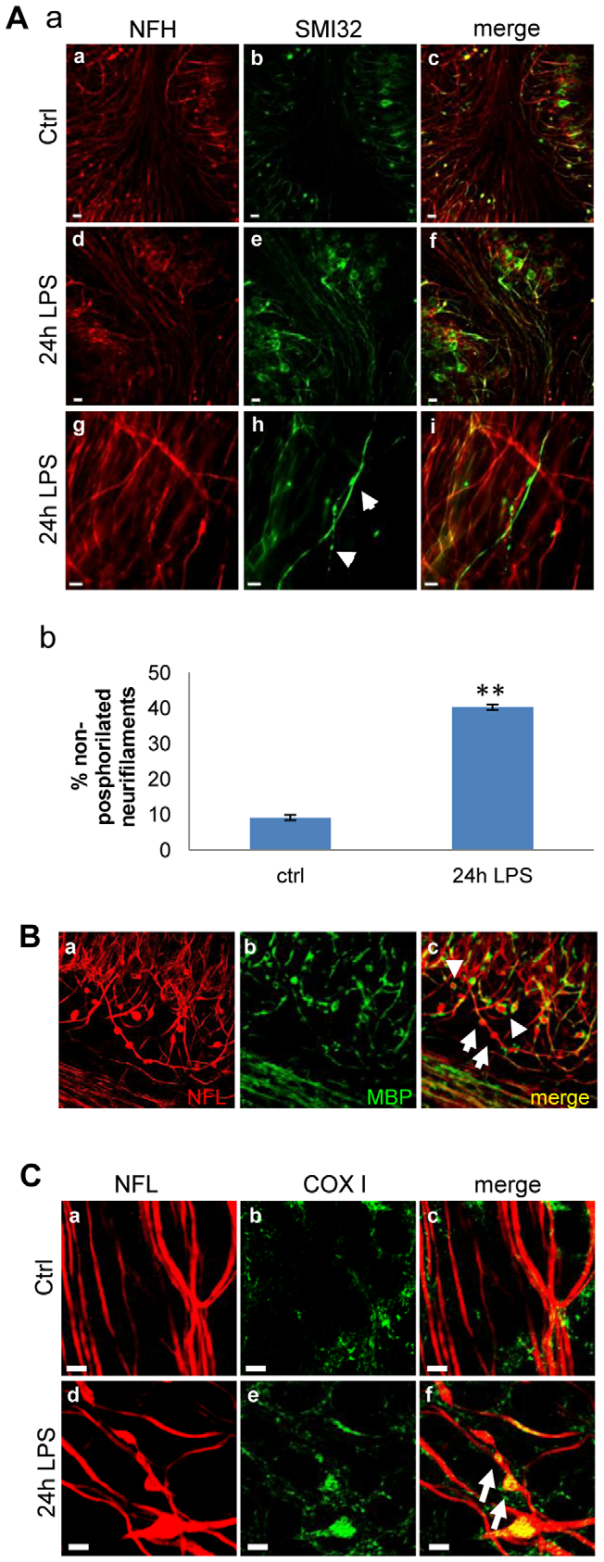
Microglial activation induces axonal damage in mouse cerebellar cultures. A) Immunostaining (a) for total neurofilament-heavy (NfH; red) and dephosphorylated NfH (SMI32; green). Panels a-c show control slices (Ctrl) while LPS treated slices are shown in panels d-i. Panels g-i show higher magnification images of d-f. Scale bar  = 20 µm (panels a-f) and 10 µm (panels g-i). Graph (b): percentage of non-phosphorylated neurofilament with respect to total neurofilaments in cerebellar cultures stimulated for 24 h with LPS. Error bars indicate the standard error. **P<0.01. Student's *t-*test was used to determine statistical significance.B) Immunostaining for NfL (red) and MBP (green) in the same conditions as in A. Arrows indicate axonal beads and arrowheads indicate axonal transection (end-bulbs). Scale bar  = 5 µm. C) Immunostaining for NfL (red) and Complex IV subunit-I (COX I, green): Neurofilament staining revealed the presence of axonal beads. Arrows indicate mitochondrial accumulation in neurofilaments. Scale bar  = 5 µm.

**Figure 4:**
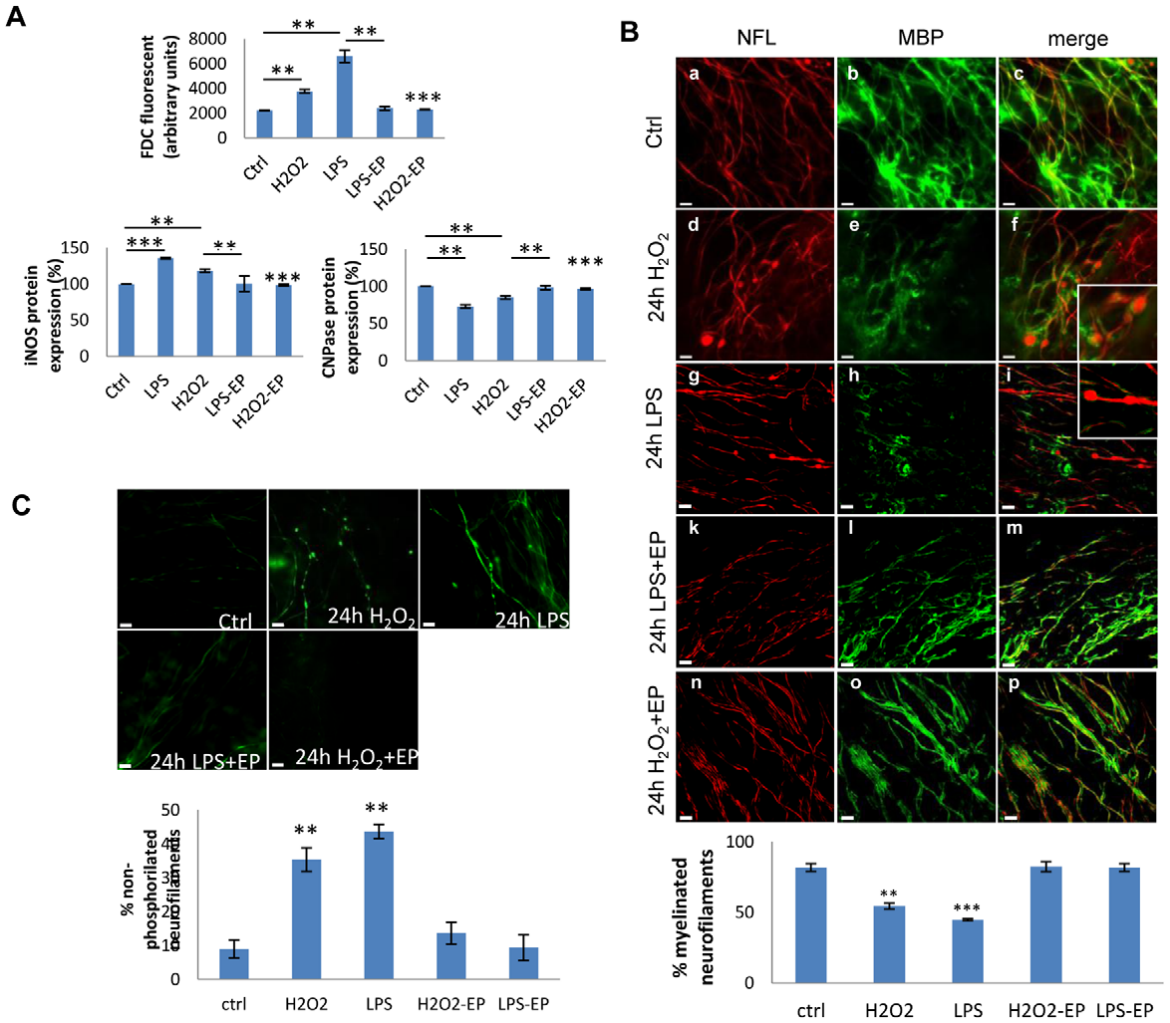
Role of Ethyl pyruvate in preventing microglia activation in cerebellar cultures. A) Comparative effects of LPS, H_2_O_2_ and Ethyl pyruvate (EP) in ROS production, iNOS expression and CNPs levels: Cerebellar cultures were left untreated (Ctrl) or treated with H_2_O_2_ (1 mM), LPS (15 µg/ml), LPS plus EP (10 mM), or H_2_O_2_ plus EP. ROS were measured by spectrofluorometry using H_2_DCFDA and expressed as arbitrary units. Total protein was extracted and analyzed in Western blots probed for iNOS and CNPase. The bands were quantified by densitometry, normalized to the total protein and expressed as a percentage with respect to the control. Error bars indicate the standard error. *P<0.05, **P<0.01 and ***P. B) Comparative effects of LPS, H_2_O_2_ and EP in demyelination and axonal damage in cerebellar cultures: Immunostaining for NfL (red, panels a, d, g, k and n) and MBP (green, panels b, e, h, l and o) in untreated organotypic cultures (control; panels a-c) or those treated for 24 h with H_2_O_2_ (panels d-f), LPS (panels g-i), LPS plus EP (panels k-m) or H_2_O_2_ plus EP (panels n-p). Co-localization is shown in the merged panels c, f, i, m and p. Insets show a higher magnification of the areas in panels f and i. Scale bar  = 10 µm. The graphic below was shows the percentage of myelinated neurofilament. Asterisks indicate the standard error calculated respect to the control. **P<0.01 and ***P. C) Role of LPS, H_2_O_2_ and EP in presence of non-phosphorilated neurofilaments (SMI32): SMI32 (green) staining in the same conditions as in B. Arrows indicates SMI32 accumulation in axons. Scale bar  = 10 µm. The graph below shows the percentage of non-phosphorylated neurofilament with respect to total neurofilaments in cerebellar cultures stimulated for 24 hrs with LPS. Asterisks indicate the standard error calculated respect to the control (statistical analysis was performed using ANOVA test) **P<0.01.

**Figure 5:**
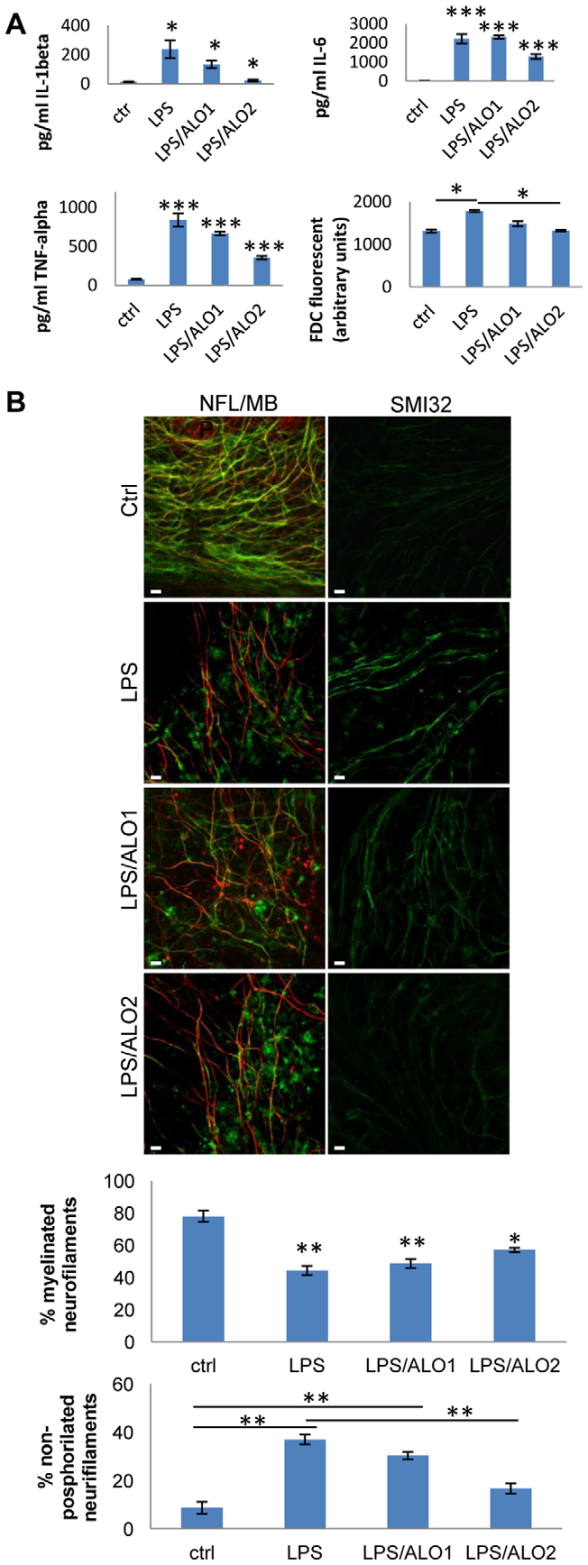
Effects of allopurinol in microglia mediated axonal damage and demyelination. A) Comparative effect of LPS and allopurinol (ALO) in cytokine expression, and ROS production by cultures: cerebellar cultures were treated with LPS in presence or absence of ALO (ALO1: 100 µM or ALO2: 1 mM). At 24 h ROS were measured and expressed as arbitrary units and IL-1β, TNF-α and IL6 release were measured by ELISA. Asterisks indicate the standard error calculated respect to the control. *P<0.05, and ***P. B) Comparative effect of LPS and allopurinol in the induction of axonal damage (non-phosphorilated neurofilaments: Immunostaining for NFL/MBP and non-phosporilated neurofilaments (SMI32) in cultures using the same condition as in D. Scale bar  = 10 µm. The graph below shows the quantification of demyelinated and non-phosporilated neurofilamentes. Error bars indicate the standard error. *P<0.05, **P<0.01 (ANOVA test).

**Figure 6:**
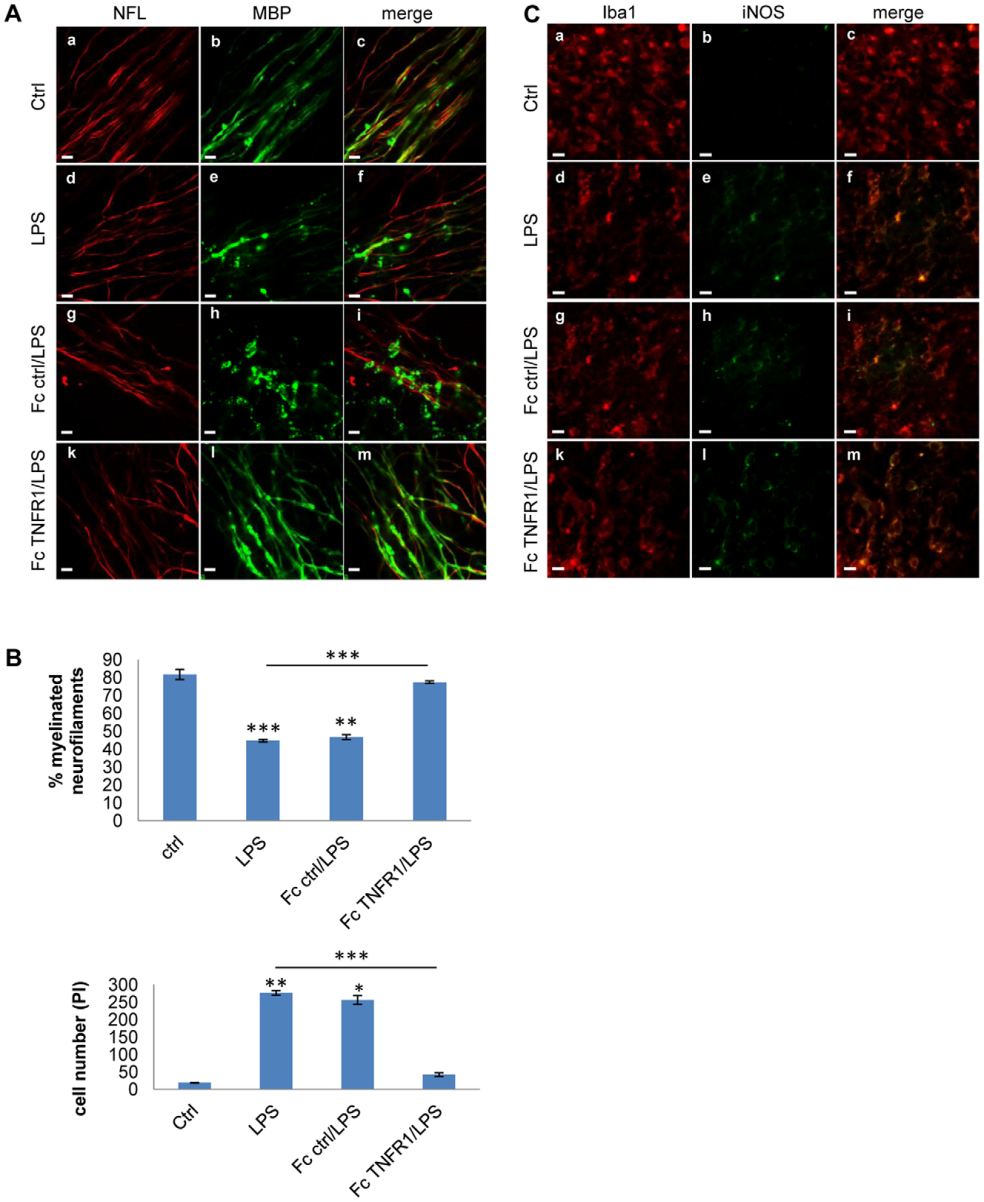
TNF-α blockade modulates microglia activation and demyelination. A) Role of TNFα blockade after LPS stimulation in demyelination of cerebellar cultures: Immunofluorescence for NfL (red) and MBP (green) in cultures untreated (ctrl, panels a-c), cultures treated with LPS (panels d-f), LPS plus control Fc (panels g-i) or LPS plus Fc-TNFR1 (15 µg/ml, panels k-m) for 24 h,. Scale bar  = 5 µm B) The graph shows the percentage of demyelinated neurofilaments (upper graph) and the number of death oligodendrocytes (PI/MBP-positive cells) (botton graph). Asterisks indicate the standard error calculated respect to the control or LPS-treated cultures. *P<0.05, **P<0.01 and ***P<0.001 (ANOVA test). C) Role of TNF-α blockade in microglia activation: Immunostaining for Iba1 (red) and iNOS (green) in the same condition as in A. Scale bar  = 5 µm.

**Figure 7:**
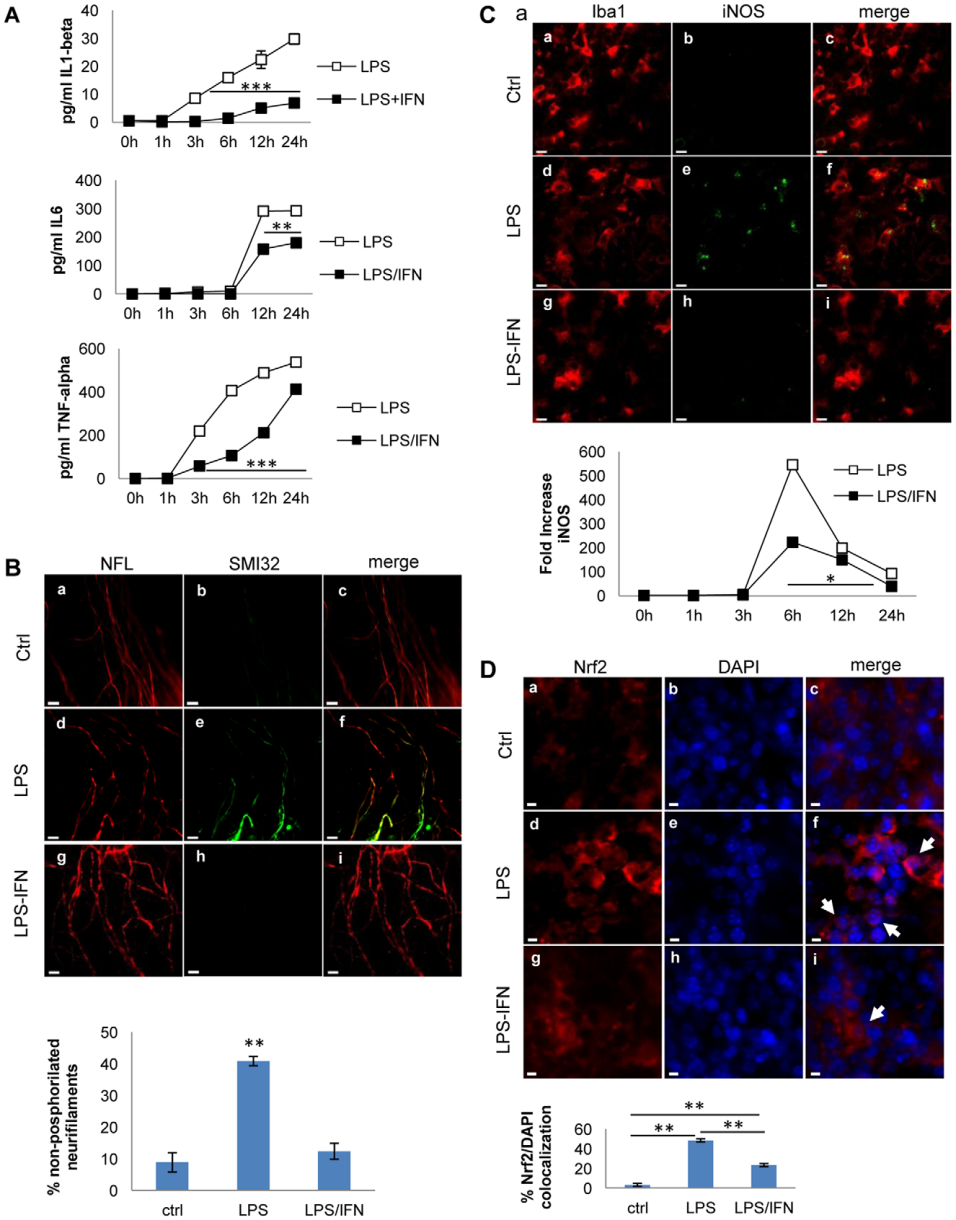
IFN-beta decreases microglia activation, cytokine release, oxidative stress and prevents axonal damage. A) IL-1β, TNF-α and IL-6 release in cerebellar cultures. Slices were treated with IFN-β for 24 h and then stimulated with LPS (15 µg/ml) for different periods of time (0, 1, 3, 6, 12, 24 h). IL-1β, TNF-α and IL-6 were quantified by ELISA. Cytokine release into the medium is expressed as pg/ml and the error bars indicate the standard error. **P<0.01 and ***P<0.001. B) Effects of IFN-β in LPS induced axonal damage: Immunostaining for NfH (red) and SMI32 (green) in cultures without LPS treatment (ctrl panels a-c), treated with LPS (panels d-f), or LPS plus IFN-β for 24 h (panels g-i). Scale bar  = 10 µm. The graph below shows the percentage of non-phosphorylated neurofilament with respect to total neurofilaments in cultures stimulated with LPS and treated with IFN-β. C) Effects of IFN-β in microglia activation and iNOS expression: Immunofluorescence staining for Iba1 (red) and iNOS (green) in the same conditions as B). iNOS levels were quantified by qPCR from cultures treated with LPS or LPS plus IFN-β: the graphs shown the fold increase over the basal values (−), normalized to the expression of the HPRT1 housekeeping gene. Error bars indicate the standard error. *P<0.05. D) Effects of IFN-β in Nrf2 nuclear translocation: Immunostaining for Nrf2 (red) and DAPI (blue) in cultures without LPS treatment (ctrl, panels a-c), treated with LPS (panels d-f), or LPS plus IFN-β for 24 h (panels g-i). Arrows indicate Nrf2 accumulation in the nucleus. Representative images of double staining are shown. Error bars indicate the standard error. **P<0.01. Scale bar  = 5 µm. ANOVA test was used to determine statistical significance.

All experiments were performed at least three times, and control cultures were time-matched with testing cultures. The values were expressed as the means ± SEM. The Student's *t-*tests (in Fig. 1, 2, 3 and Supp. Fig. S1 and S3) or ANOVA (in Fig. 4, 5, 6 and 7) were used to determine statistical significance and all analyses were performed using SPSS 15.0 software (IBM).

## Results

### LPS induces microglia activation in mice cerebellar organotypic cultures

In order to reproduce microglia activation as one of the mechanisms present in neuroinflammation, cerebellar organotypic cultures were challenged with LPS. First, we determined the concentration and timing of LPS needed to induce microglia activation by treating the cultures for 24 h with different concentration of LPS (5, 10, 15 and 20 µg/ml), and analyzing the kinetics of IL-1β, TNF-α and IL-6 release over 96 h by ELISA (Supp. Fig. S1). When cultures were challenged with doses up to 15 µg/ml, LPS induced a dose-dependent production of pro-inflammatory cytokines, such as IL-1β (Supp. Fig. S1A). Peak levels of secreted TNF-α, IL-6 and IL-1β were observed 3, 12 and 24 h after LPS challenge, respectively. While the release of IL-1β was transient, constant high levels of TNF-α and IL-6 were secreted from 3 h up to the last measurement at 96 h (approximately 500 pg/ml and 1800 pg/ml, respectively) (Supp. Fig. S1B). To assess microglia activation, organotypic cultures were stained with markers of activated microglia such as MHCII and OX42 [14]. We observed the presence of amoeboid shaped microglial cells, with enhanced MHC-II and OX42 expression 24 h after LPS challenge (Supp. Fig. S1C), both features indicative of microglial activation. We did not observe MHC-II and OX42 positive cells in the control cultures at the same time point (data not shown).

### Activation of microglia by LPS induces demyelination and oligodendrocyte death

To determine whether neuroinflammation induces demyelination in the cerebellar organotypic model, we analyzed CNPase protein expression in Western-blot. Cultures were grown for 7 DIV to allow significant myelination and then exposed to LPS (15 µg/ml) for 1, 3, 6, 12, 24, 48, 72 and 96 h, which produced a 40% loss of CNPase protein after 96 h, which had fallen significantly by 24 h (Fig. 1A). Slices were also counterstained with NfL and MBP antibodies (Fig. 1B) for different times after LPS treatment. We observed significant demyelination at 24 h, revealed as a decrease in the intensity of myelin immunoreactivity at different magnifications that was maintained until 96 h (Fig. 1B and Supp. Fig. S2). The 24 h time point was therefore used as reference in all subsequent demyelination experiments. Confocal analysis revealed only a few myelinated axons (Fig. 1B, panel m) in LPS-challenged cultures when compared to time-matched controls. In cultures treated with LPS, the myelin staining appeared punctuate and sharp, a hallmark of myelin damage. CNPase staining revealed a similar profile of demyelination as MBP staining (data not shown). In response to LPS treatment there was an approximately 2-fold decrease in MBP staining compared to time-matched control tissue (Fig. 1B, graph). Demyelination after LPS challenge was confirmed by electron microscopy (Fig. 1C).

To determine whether LPS stimulation induced the cell death, cultures were exposed to LPS (15 µg/ml) for 24 h, and stained for activated (cleaved) caspase 3 (Fig. 1D and Supp. Fig. S3B). LPS treatment enhanced glia cell death that was mainly involving oligodendrocytes, revealed by an increased number of MBP/Casp3-positive cells (Fig. 1D). Evidence of oligodendrocytes cell death was also obtained when the cultures were labeled with propidium iodide (PI) for the last 2 h, revealing an increased number of MBP/PI-positive cells (Supp Fig. S3A), although some other glial cell types cannot be excluded. In contrast, only a few neurons died in the grey matter, as revealed by NeuN/Casp3, NeuN/PI and Hoechst/Casp3 co-labeling (Fig. 1D and Supp. Fig. S3A and B). These findings indicate that exposure to LPS induced significant cell death in the white matter, and is consistent with oligodendrocyte cell death seen in the LPS model of optic nerve injury [15].

### LPS induces oxidative stress and axonal damage in mouse cerebellar cultures

To analyze whether LPS induce oxidative stress in mice organotypic cultures, we quantified iNOS expression and ROS production at different times after LPS challenge (0 to 96 h, Fig. 2). Western blot analysis showed that LPS increased levels of iNOS protein peaking at 12 to 24 h after challenge and decreased subsequently up to the study end-point (96 h, Fig. 2A). Furthermore, using fluorescent assay with H_2_DCFDA we observed a 100% and 40% increase in ROS production 12 h and 24 h after the LPS challenge, respectively (Fig. 2B). To verify whether the actual producer cells of iNOS were microglia or astrocytes, the cultures were immunostained for iNOS and the microglial marker Iba1 after LPS stimulation. As expected, in the slices treated with LPS, iNOS was mainly expressed by microglia cells (Fig. 2C).

Presence of axonal damage was assessed by double immunostaining for both total (phosphorylated and non-phosphorylated) NfH and non-phosphorylated NfH (SMI32; Fig. 3A). In response to LPS treatment, non-phosphorylated NfH was found to accumulate in the neurofilaments (arrows in Fig. 3A panel h) with a 4-fold increase at 24 h compared to total NfH, suggesting presence of axonal dysfunction (Fig. 3A, panel b). Furthermore, axonal dysfunction was visible in slices challenged with LPS by means of immunostaining for NfL and MBP, showing the formation of swollen structures (beading or spheroids; arrows in Fig. 3B, panel c and Supp. Fig. S2) indicating impaired axonal transport, as well as with axonal transection (end-bulbs; arrowheads in Fig. 3A, panel c and Supp. Fig. S2) [1]. Based on our results showing maximum axonal damage by 24 h after LPS challenge, this time point was used for assessing axonal damage. Finally, we analyzed the changes in the distribution of axonal mitochondria by staining the respiratory chain complex IV subunit-I (COX-I) after stimulation with LPS (15 µg/ml) for 24 h (Fig. 3 C). We observed an accumulation of COX-I labeled mitochondria in the spherical axon bulbs, indicative of altered mitochondrial transport (Fig. 3C, arrows in panel f). No such accumulation of mitochondria was observed in the time-matched control cultures (Fig. 3C, panel c).

### Contribution of oxidative stress to axonal and myelin damage

To assess the contribution of oxidative stress to axonal damage, we compared the effect of LPS induced oxidative stress with that induced by hydrogen peroxide (H_2_O_2_), a promoter of free radicals, in the cerebellar culture model. ROS production induced by LPS after 24 h was 3-fold higher than that in time-matched control slices and 2-fold higher than that induced by a low dose of H_2_O_2_. Indeed, LPS induced a 36% and 15% increase in iNOS protein expression with respect to control slices and those treated with a low dose of H_2_O_2_ (Fig. 4A). Furthermore, demyelination was evident in both LPS and H_2_O_2_-treated samples, as detected in CNPase Western-blots (Fig. 4A), and by immunofluorescence for NfL and MBP (Fig. 4B). The extensive loss of myelin generated by LPS treatment was associated with greater axonal swelling than in control or H_2_O_2_–treated samples (Fig 4B panels f and i). Axonal damage was greater 24 h after the LPS challenge when compared with H_2_O_2_ treatment, as determined by specific staining for anti-non-phosphorylated NfH (Fig. 4C).

### The microglia activation inhibitors ethyl pyruvate and allopurinol decreased demyelination and axonal damage

To study the effect of microglia activation on axonal damage and demyelination in this model, we tested the effect of the iNOS inhibitor ethyl pyruvate (EP) [16], [17]. EP is a stable form of pyruvate, a metabolite with strong anti-oxidant and scavenger activity, which inhibits expression of iNOS. EP inhibits JAK2 phosphorylation, which in turn inhibits the phosphorylation of STAT1 and STAT3 in LPS-stimulated microglia and as a consequence, suppresses the expression of the STAT-responsive genes iNOS and cyclooxygenase-2. In addition, EP reduces LPS-induced ROS production by inhibiting gp91phox transcription and Rac1 activity, suppressing the Rac1-JAK-STAT signaling cascade [16]. Figure 4A shows that in the presence of EP, ROS production induced by LPS returned to levels similar to those of untreated controls, which was associated with decreased iNOS activation. Axonal damage, as determined by the increase in non-phosphorylated NfH, was also reduced to control levels in the presence of EP (Fig. 4C). Moreover, CNPase or MBP protein levels were preserved by EP treatment (Fig. 4A and 4B). In summary, EP decreased demyelination and axonal damage due to the inhibition of microglia activation.

Second, we treated LPS challenged cultures with the xanthine oxidase inhibitor and radical-free scavenger Allopurinol [18]. This compound is a ROS scavenger that does not affect MAPK activation in microglia [18]. We tested different concentration of Allopurinol in the microglia cell line BV2 treated with LPS and measured the release of pro-inflammatory cytokines and ROS production. Allopurinol significantly decreased ROS levels without significantly modifying IL-1β, IL-6 and TNF-α secretion (data not shown). Cerebellar cultures were pretreated for 2 h with allopurinol using two different concentrations (100 µM and 1 mM) and then stimulated with 15 µg/ml of LPS for 24 h. We found a significant ROS decrease as quantified by H_2_DCFDA assay in the cultures treated with allopurinol 1 mM after LPS challenge compared with time-matched cultures stimulated with LPS (Fig. 5A). Moreover, to verify that allopurinol does not interfere with microglia activation we tested IL-1β, IL-6 and TNF-α release by ELISA assay. Allopurinol was not able to block cytokine release induced by LPS to a significant extent when we treated the cultures with 100 µM of allopurinol. In contrast, allopurinol blocks cytokine release at 1 mM (Fig. 5A). However, when we compared IL-1β, IL-6 and TNF-α levels from cultures treated with allopurinol (1 mM) after LPS challenge with time-matched control cultures (without LPS) we found a significant increase of cytokines release (Fig. 5A). These results suggest that allopurinol was not able to block microglia activation completely, even if it did block ROS production. Finally, we assessed the effect of microglia activation modulated by allopurinol on demyelination and axonal damage. After 24 h of treatment with LPS in presence or absence of allopurinol, cultures were stained for neurofilament light (NfL) and MBP (Fig. 5B, left panels). Allopurinol used at 1 mM significantly prevented axonal damage (Fig. 5B, right panels) but did not decrease demyelination (Fig. 5B, see quantification in graphs below).

### Blocking TNF-α prevents partially demyelination but not oxidative stress-mediated axonal damage

During brain inflammation, pro-inflammatory cytokines and oxidative stress may differentially contribute to axon and myelin damage. To investigate the contribution of pro-inflammatory cytokines such as TNF-α to tissue damage, TNF-α activity was inhibited using a blocking recombinant protein (Fc-TNFR1), and the levels of oxidative stress and axonal and myelin damage in cultures were measured. Demyelination was significantly attenuated in cerebellar cultures pretreated with Fc-TNFR1 2 h before the LPS challenge (Fig. 6A), visible as a significant increase in the percentage of myelinated axons in the Fc-TNFR1 group compared to the LPS group (Fig. 6B). We quantified oligodendrocyte cell death by double staining with MBP/PI. We observed that Fc-TNFR1 treated cultures had a significantly decrease of oligodendrocyte death compared to the cultures treated with LPS (Fig. 6B). These effects were present without modification of iNOS expression (Fig. 6C). In summary, in the cerebellar culture model of neuroinflammation, myelin damage and oligodendrocyte loss were promoted by TNF-α.

### Role of interferon-beta therapy in preventing oxidative stress-mediated axonal damage

Interferon-beta (IFN-β) is the most common treatment for MS, with a pleiotropic mechanism of action, preventing CNS damage. However, the precise role of IFN-β in controlling oxidative stress in MS is uncertain, particularly given that type I IFN activates iNOS in monocytes and promotes ROS generation [19], [20], while it can also downregulate iNOS expression in other settings [21]. First, we assessed the effects of IFN-β in the release of proinflammatory cytokines by LPS. Cytokine release was significantly attenuated in presence of IFN-β (Fig. 7A). Specifically, IFN-β has a more profound and early effect on IL-1β than on IL-6 and TNF-α release. Furthermore, cultures treated with IFN-β had significantly less axonal damage, as revealed by a reduction in the percentage of non-phosphorylated neurofilaments in cultures treated with IFN-β after LPS challenge (Fig. 7B).

In order to assess the effect of IFN-β on oxidative stress, we analyzed iNOS and Nrf2 expression. Pretreatment with IFN-β prior to the LPS challenge reduced LPS-induced iNOS expression, as determined both by RT-PCR (Fig. 7C) and by increasing the protein levels in the tissue and translocation to nucleus (Fig. 7D). Nrf2 is a transcription factor that regulates the expression of many phase II detoxifying and antioxidant enzymes. The increase of Nrf2 is a molecular sensor of oxidative stress and its decrease would suggest reduced oxidative stress. Thus, we observed that LPS-induced oxidative stress triggers translocation of Nrf2 in the nucleus, and that IFN-β treatment induced 50% decrease in Nrf2 translocation (Fig. 7D). Taken together, these results indicate that IFN-β displays an anti-oxidant and anti-inflammatory role in the mice cerebellar model and also highlights the usefulness of this model for monitoring the effects of MS therapies.

## Discussion

The LPS model of neuroinflammation in cerebellar cultures [22] recapitulates several events that occur during brain inflammation, including microglia activation followed by cytokine release and oxidative stress, demyelination and axonal damage. Using this model we have evaluated the effect of microglia activation on demyelination and axonal damage in cerebellum tissue. Moreover, we have analyzed whether the murine organotypic culture model represents an effective tool to study the effects of drugs used in neuroinflammatory diseases by using IFN-β as an example. Our results indicate that LPS induced microglia activation in organotypic cultures, as observed by presence of microglial cells with amoeboid shape that expressed MHC-II and OX42, the release of pro-inflammatory cytokines, such as IL-1β, IL-6 and TNF-α and the induction of oxidative stress. Microglia activation was associated with oligodendrocyte death and myelin and axonal damage. Demyelination occurs in cerebellar cultures challenged with LPS, although to a lesser extent than in models of demyelination induced by lysolecithin [23], passive transfer of anti-MOG antibodies in cerebellar cultures [24], or LPS challenge to optic nerve cultures [15]. We need to keep in mind that cerebellar tissue appears to be more sensitive to oxidative damage than other brain regions [25]. Cerebellar cultures preserve to a large extent the structure of the brain tissue, and all the cell populations of interest (microglia, astrocytes, neurons and axons, myelin and oligodendrocytes) when compared with spinal cord, retina or hippocampus cultures. The analysis of the effects of neuroinflammation elicited by LPS facilitates dissection of the pathogenic process present in brain inflammatory diseases. *In vivo*, LPS injection in the spinal cord has been shown to induce significant immune cell recruitment to the site of injection, with prominent demyelination that develops over 2 weeks and to a lesser extent axonal damage, followed by remyelination by Schwann cells 4 weeks later [26]. Although the hematogenous inflammation typical of MS and other inflammatory brain diseases does not develop in this model, the effect of activating the innate immune system within the brain is recapitulated by the presence of microglia activation, which appears to be critical for the long-term axonal damage in MS and degenerative diseases [27].

In the cerebellar cultures stimulated with LPS, we observed ROS production and iNOS expression in activated microglia indicating induction of oxidative stress. LPS activates microglia and astrocytes by binding to TLR4, promoting the induction of iNOS, which in turn produces ROS [28]. Activation of microglia and astrocytes occurs at different stage in several neurodegenerative diseases. In experimental autoimmune encephalomyelitis (EAE), microglia proliferate at the initial stage while astrocytes start to respond more markedly at the late recovery stage [29]. In general, activated astrocytes also express iNOS and the levels of iNOS observed in the organotypic cultures challenged with LPS probably also depend on astrocytes. Oligodendrocytes and myelin are highly sensitive to NO, which provokes the deregulation of the mitochondrial electron transport chain in association with the translocation of the apoptosis inducing factor (AIF) [30] and the production of peroxynitrite [31]. The reduction, but not complete suppression, of demyelination by iNOS inhibitors suggests that oligodendrocytes are damaged by other mechanisms triggered through the activation of TLR4 by LPS. Indeed, TNF-α and IL-1β appear to mediate oligodendrocyte damage in mixed cultures [15], [32]. The requirement of a mixed glia environment suggests that cytokines impair the glutamate-buffering capacity of astrocytes [33].

To evaluate the contribution of microglia activation on demyelination and axonal degeneration we also may use of chemical inhibitors of microglia activation such as EP or allopurinol. Axonal damage was elicited by LPS-mediated microglia activation as well as by H_2_O_2_-promoted oxidative stress. Inhibition of iNOS expression by EP prevented myelin and axonal damage whereas allopurinol preferentially prevented axonal loss, but demyelination persists. In particular, allopurinol reduced significantly the production of ROS and slightly the amount of cytokines. The amount of cytokines still present after allopurinol pre-treatment (>500 pg/ml for TNF-α, 100 pg/ml for IL-6 and 50 pg/ml for IL-1β) is sufficient to induce demyelination in the cultures. Moreover, in the present model we found that following inhibition of TNF-α, myelin damage and oligodendrocyte loss were promoted by pro-inflammatory cytokines. However, we did not blocked other pro-inflammatory cytokines and for this reason we cannot rule out the contribution of other pro-inflammatory cytokines to tissue damage.

Oxidative stress may contribute to axonal damage via several mechanisms, including the impairment of mitochondrial function due to the accumulation of mutations in mtDNA. In turn, this leads to energetic failure, protein and lipid oxidation, and microtubule degradation, thus impairing functions such as axonal transport and structural support [1], [3], [34]. The axonal swelling and mitochondria accumulation were pertinently present in the model and were consistent with a disruption of microtubules by oxidative stress and the subsequent blockade of axonal transport [6]. Moreover, demyelination enhances this effect due to the lack of metabolic support provided by myelin in long axons [35], [36].

Finally we have investigated the effect of IFN-β, a firmly established first-line therapeutic agent for MS that prevents CNS damage. Our model indicates that IFN-β decrease both the expression of pro-inflammatory cytokines and oxidative stress, as such contributing to axonal preservation. The occurrence of oxidative damage coincided with increased levels of antioxidant enzymes in cerebellar cultures treated with IFN-β, suggesting that the antioxidant capacity is overwhelmed during a neuroinflammatory attack [37]. Several studies have also shown that IFN-β inhibits cytokine-induced NO- or iNOS-synthesis in astrocytes, which may contribute to its clinical efficacy [21], [38], [39]. In contrast, Lieb et al [40] have shown no inhibitory effects of IFN-β on iNOS in rat microglial cells. Moreover, studies in murine macrophages have shown that IFN-β increased the iNOS activity, thereby enhancing intracellular NO activity [20]. In our culture system, IFN-β reduced iNOS expression supporting the protective effect of IFN-β from oxidative stress. Endogenous antioxidant enzymes are regulated by the transcription factor Nrf2 and upon exposure to ROS, Nrf2 translocates to the nucleus where it binds to antioxidant response elements in genes coding for antioxidant enzymes [41]. In organotypic cultures challenged with LPS, translocation of Nrf2 into the nucleus was decreased when the cultures were treated with IFN-β. We hypothesize that the decrease in nuclear translocation of Nrf2 observed is probably due to the ability of IFN-β to prevent oxidative stress.

In summary, our data underscore that in cerebellar organotypic cultures challenged with LPS, microglial activation is sufficient to release pro-inflammatory cytokines and induce oxidative stress, damaging myelin and axons, even in the absence of lymphocytes and hematogenous macrophages. These events were recapitulated and dissected in this model, providing a tool to study tissue damage MS and other inflammatory brain diseases, that might reflect the progression of the disease in the absence of overt inflammatory infiltrates. Moreover, this model permits the mechanistic study of new treatments for MS and other neuroinflammatory disorders.

## Supporting Information

Figure S1 **LPS induces microglia activation in mouse cerebellar cultures.** A) dose-response curve of IL-1β release measured by ELISA. Cerebellar cultures were stimulated with four different concentrations of LPS for 24 h (5, 10, 15 and 20 µg/ml). B) IL-1β, TNF-α and IL-6 release in cerebellar cultures. Slices were stimulated with LPS (15 µg/ml) for different periods of time (0, 1, 3, 6, 12, 24, 48, 72 and 96 h) and then analyzed by ELISA. Cytokine release into the medium is expressed as pg/ml. Error bars indicate the standard error. *P<0.05, **P<0.01 and ***P<0.001 (Student's *t*-test). C) Immunostaining for markers of microglia activation MHCII (a) and OX42 (b). Cultures were stimulated with LPS (15 µg/ml) for 24 h and then stained with antibodies against MHCII and OX42. Scale bars  = 50 µm (a) and 10 µm (b).
(TIF)

Figure S2 **Demyelination and impaired axonal transport** **were maintained after 24** **h of LPS treatment.** Cerebellar cultures were stimulated with LPS (15 µg/ml) for 0, 1, 3, 6, 12, 24, 48, 72 and 96 h, and stained for NfH (red) and MBP (green). The time points 12 h and 96 h after LPS challenge are shwon. Arrows indicate axonal beads and arrowheads indicate axonal transection (end-bulbs). Scale bar  = 10 µm.
(TIF)

Figure S3 **Microglial activation induces oligodendrocyte death in mouse cerebellar cultures.** A) Cerebellar cultures were treated with LPS (15 µg/ml) for 24 h and then immunostained for MBP (green) or NeuN (blue) and counterstained with propidium iodide (red). The graph shows the number of PI-MBP-positive cells. Higher magnification images of white (g) and grey (h) matter in cultures treated with LPS. Scale bar  = 100 µm (panels a-f) and 10 µm (panels g and h). B) Cerebellar cultures were incubated for 24 h in the presence (LPS) or absence (Ctrl) of LPS (15 µg/ml). Immunocytochemistry was performed to detect activated (cleaved) caspase-3 labeling. Graphs show the percentage of cell death by quantifying the co-localization of active Casp3 immunofluorescence in conjunction with Hoechst 33258 nuclear staining. Asterisk indicates the standard error calculated respect to the control. *P<0.05, **P<0.01 (Student's *t*-test). Representative images of double staining for active Casp3 (green) and Hoechst (blue) are shown. Scale bar  =  5 µm.
(TIF)
