# Supplementary figures and images for: Oxidative Stress and Proinflammatory Cytokines Contribute to Demyelination and Axonal Damage in a Cerebellar Culture Model of Neuroinflammation
Source: PLoS One. 2013 Feb 19;8(2):e54722. doi: 10.1371/journal.pone.0054722 (PMC3576396; doi:10.1371/journal.pone.0054722)

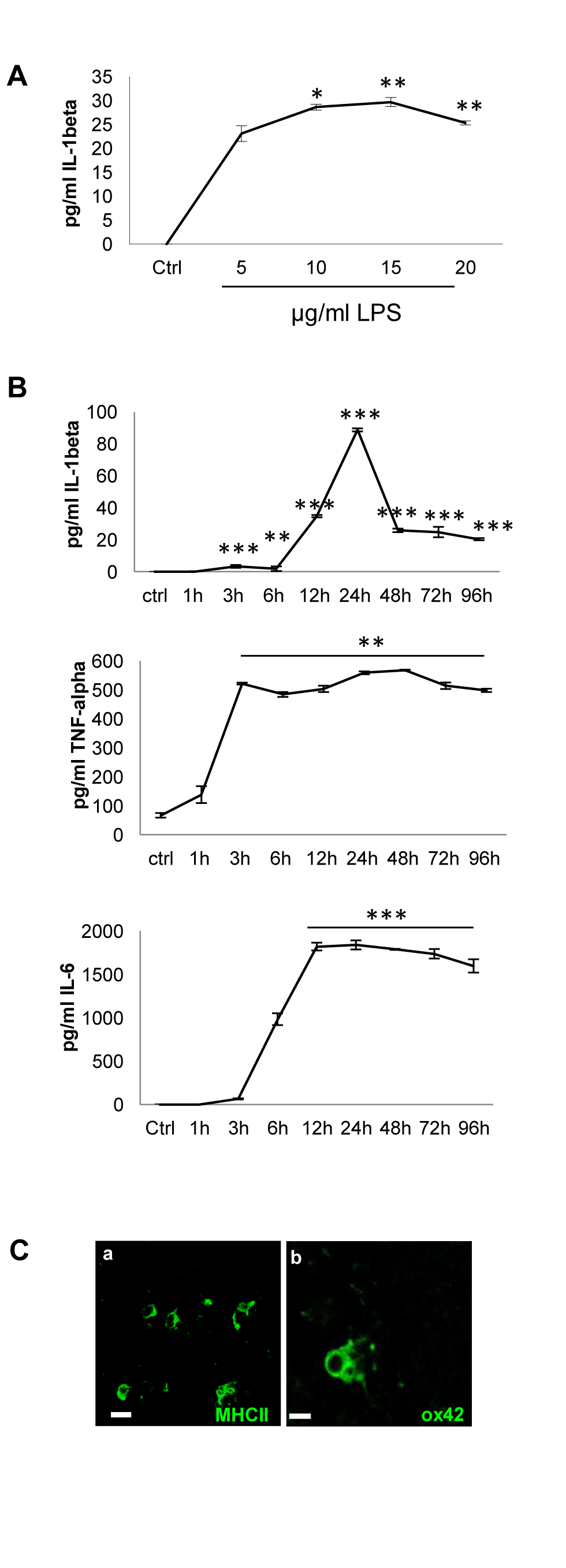

Supplement: Figure S1 — LPS induces microglia activation in mouse cerebellar cultures. A) dose-response curve of IL-1β release measured by ELISA. Cerebellar cultures were stimulated with four different concentrations of LPS for 24 h (5, 10, 15 and 20 µg/ml). B) IL-1β, TNF-α and IL-6 release in cerebellar cultures. Slices were stimulated with LPS (15 µg/ml) for different periods of time (0, 1, 3, 6, 12, 24, 48, 72 and 96 h) and then analyzed by ELISA. Cytokine release into the medium is expressed as pg/ml. Error bars indicate the standard error. *P<0.05, **P<0.01 and ***P<0.001 (Student's t-test). C) Immunostaining for markers of microglia activation MHCII (a) and OX42 (b). Cultures were stimulated with LPS (15 µg/ml) for 24 h and then stained with antibodies against MHCII and OX42. Scale bars = 50 µm (a) and 10 µm (b). (TIF) [file pone.0054722.s001.tif]

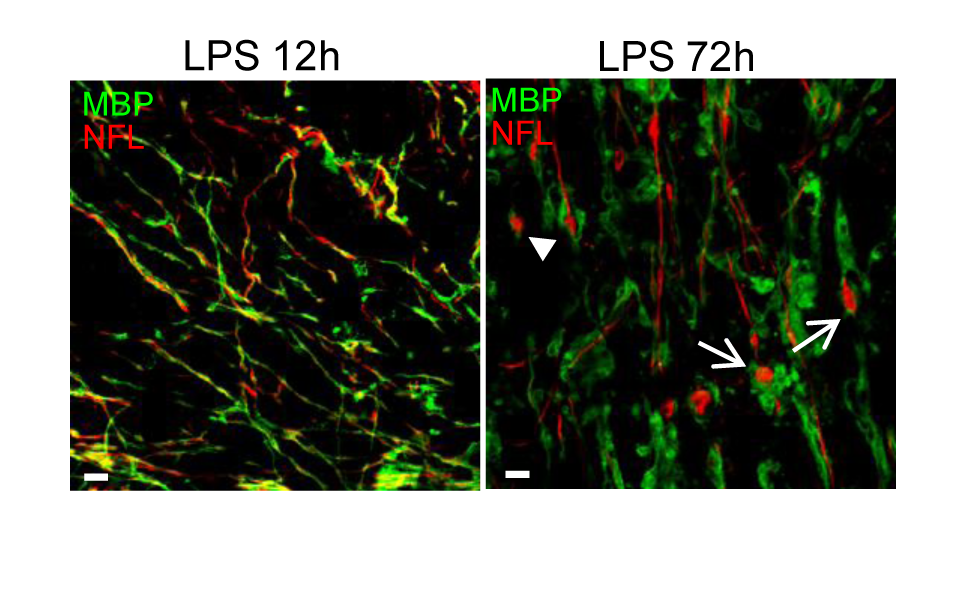

Supplement: Figure S2 — Demyelination and impaired axonal transport were maintained after 24 h of LPS treatment. Cerebellar cultures were stimulated with LPS (15 µg/ml) for 0, 1, 3, 6, 12, 24, 48, 72 and 96 h, and stained for NfH (red) and MBP (green). The time points 12 h and 96 h after LPS challenge are shwon. Arrows indicate axonal beads and arrowheads indicate axonal transection (end-bulbs). Scale bar = 10 µm. (TIF) [file pone.0054722.s002.tif]

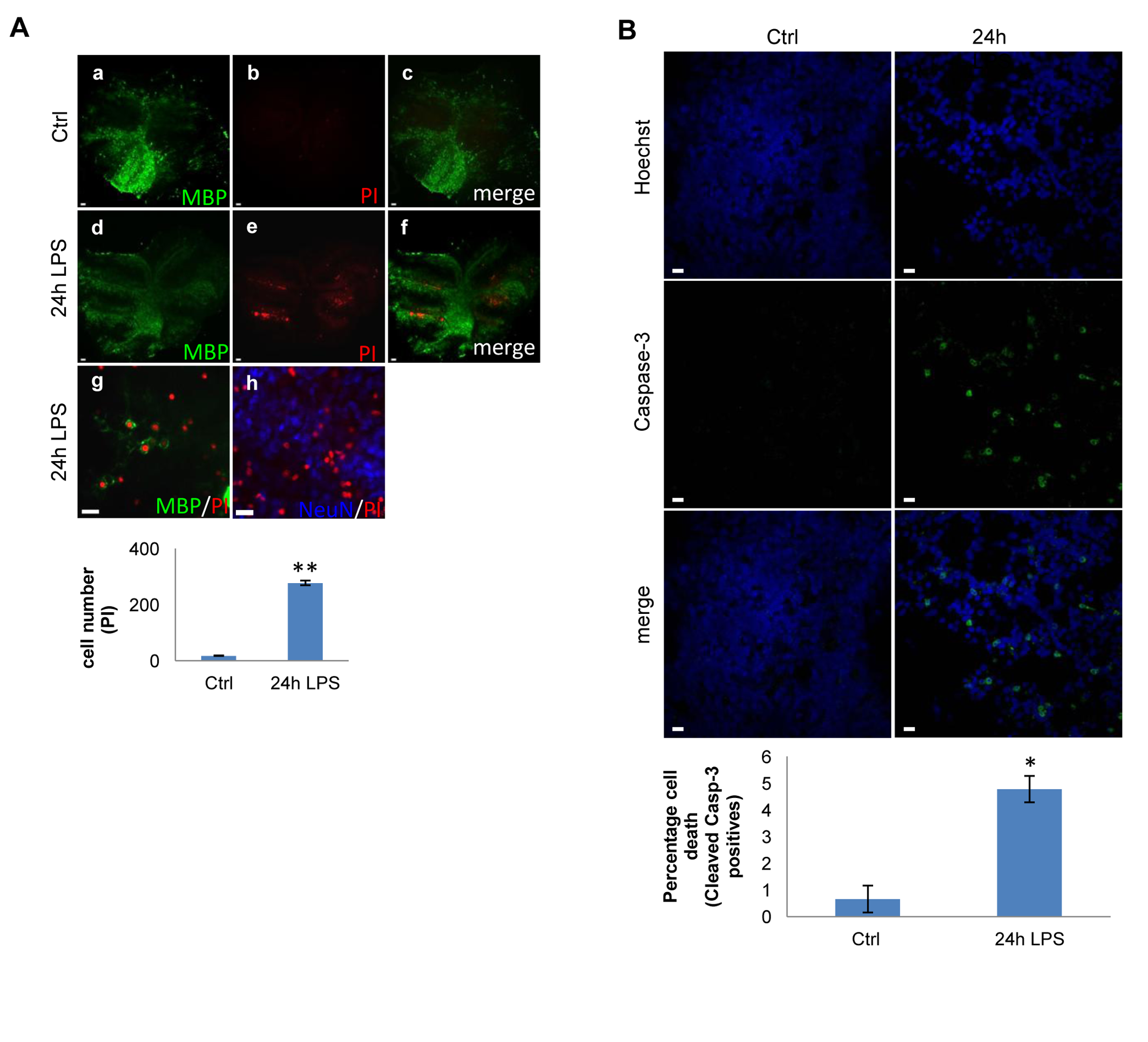

Supplement: Figure S3 — Microglial activation induces oligodendrocyte death in mouse cerebellar cultures. A) Cerebellar cultures were treated with LPS (15 µg/ml) for 24 h and then immunostained for MBP (green) or NeuN (blue) and counterstained with propidium iodide (red). The graph shows the number of PI-MBP-positive cells. Higher magnification images of white (g) and grey (h) matter in cultures treated with LPS. Scale bar = 100 µm (panels a-f) and 10 µm (panels g and h). B) Cerebellar cultures were incubated for 24 h in the presence (LPS) or absence (Ctrl) of LPS (15 µg/ml). Immunocytochemistry was performed to detect activated (cleaved) caspase-3 labeling. Graphs show the percentage of cell death by quantifying the co-localization of active Casp3 immunofluorescence in conjunction with Hoechst 33258 nuclear staining. Asterisk indicates the standard error calculated respect to the control. *P<0.05, **P<0.01 (Student's t-test). Representative images of double staining for active Casp3 (green) and Hoechst (blue) are shown. Scale bar = 5 µm. (TIF) [file pone.0054722.s003.tif]
